# Association between Hydration Status and Body Composition in Healthy Adolescents from Spain

**DOI:** 10.3390/nu11112692

**Published:** 2019-11-07

**Authors:** Ana Isabel Laja García, Carmen Moráis-Moreno, Mª de Lourdes Samaniego-Vaesken, Ana M. Puga, Gregorio Varela-Moreiras, Teresa Partearroyo

**Affiliations:** Department of Pharmaceutical and Health Sciences, Universidad CEU San Pablo, 28668 Madrid, Spain; a.laja90@gmail.com (A.I.L.G.); car.morais.ce@ceindo.ceu.es (C.M.-M.); l.samaniego@ceu.es (M.d.L.S.-V.); anamaria.pugagimenezazca@ceu.es (A.M.P.); gvarela@ceu.es (G.V.-M.)

**Keywords:** water intake, water balance, weight management, obesity, overweight, body composition

## Abstract

At present, obesity and overweight are major public health concerns. Their classical determinants do not sufficiently explain the current situation and it is urgent to investigate other possible causes. In recent years, it has been suggested that water intake could have important implications for weight management. Thus, the aim of this study was to examine the effect of hydration status on body weight and composition in healthy adolescents from Spain. The study involved 372 subjects, aged 12–18 years. Water intake was assessed through the validated “hydration status questionnaire adolescent young”. Anthropometric measurements were performed according to the recommendations of the International Standards for Anthropometric Assessment (ISAK) and body composition was estimated by bioelectrical impedance analysis. Water intake normalized by body weight was positively correlated with body water content (boys (B): r = 0.316, *p* = 0.000; girls (G): r = 0.245, *p* = 0.000) and inversely with body mass index (BMI) (B: r = −0.515, *p* = 0.000; G: r = −0.385, *p* =0.000) and fat body mass (B: r = −0.306, *p* = 0.000; G: r = −0.250, *p* = 0.001). Moreover, according to BMI, overweight/obese individuals consumed less water than normal weight ones. In conclusion, higher water balance and intake seems to be related with a healthier body composition.

## 1. Introduction

Obesity and overweight are currently the fifth leading global risk factor for mortality [1]. Its continuously increasing prevalence throughout the world has made prevention a major public health challenge. The current situation in children and adolescents is an issue of special concern, given that, according to the World Health Organization (WHO), over 340 million people aged between five and nineteen years are obese or overweight [2]. Particularly in Spain, the latest National Health Survey [3] has shown that the prevalence of this chronic disease in children and adolescents has increased dramatically in the last thirty years. This situation has resulted in diseases and health problems that were previously observed only in adults, such as diabetes mellitus, arterial hypertension, coronary artery disease and/or fatty liver disease, affecting obese children [4,5,6]. Consequently, the resulting complications of these pathologies in this population group are even more severe, leading to short life and worse quality of life [4,7]. Furthermore, childhood is the best stage of life to prevent diseases and to promote healthy habits and, thus, the prevention of obesity is of major importance. 

It is well known that the main determinants of overweight and obesity are diet and physical activity; nevertheless, they do not sufficiently explain the current situation [8,9]. In recent years, several investigations have demonstrated that there are other factors implicated in weight regulation and obesity development [10,11], such as water intake and hydration status, which is defined as the body’s fluid level and is determined by water balance (net difference between water input and output) [12]. Its positive role has been documented by several studies conducted in adults [13,14,15,16,17,18] as well as in children [19,20]. In fact, some systematic reviews [14,18,19] conclude that water consumption might have a beneficial effect on weight status as well as a weight reducing effect, although the available evidence for the establishment of causal associations is still scarce. In this context, our research group has recently published a cross-sectional study [21] conducted in healthy adults, where inverse associations between water consumption (normalized by body weight) and body weight, fat body mass and waist circumference were found. Understanding the mechanisms for the aforementioned associations are of interest and although this remains unclear, it has been suggested that a combination of the effects derived from water consumption could explain them. Thus, water consumption leads to satiety increase [22,23,24], lipolysis rate and energy expenditure enhance by means of sympathetic stimulus and thermogenesis induction [25,26], caloric intake decrease due to mistakes in the perception of hunger and thirst cues [27] and improvement in diet quality [28,29,30], among others. Despite all acquired knowledge, available data of water intake for the general population and in particular for children are a cause for concern. According to data from the “anthropometric data, macronutrients and micronutrients intake, practice of physical activity, socioeconomic data and lifestyles in Spain” (ANIBES) study [31], the daily water intake from Spanish children aged between 13 and 17 years was 1398.04 ± 43.29 mL in boys and 1235.51 ± 40.07 mL in girls, thus, lower than the European Food Safety Authority (EFSA) water intake recommendations (2100 mL/day and 1900 mL/day for boys and girls aged 9–13 years, respectively) [32]. Moreover, adolescents aged 14 years and older are considered as adults regarding EFSA water intake recommendations (2500 mL/day and 2000 mL/day in males and females, respectively). This fact could entail several negative effects on the health and wellness of the target population, since a proper hydration status is essential for the adequate functioning of the human body [33,34].

For all the aforementioned, novel studies that confirm and allow deepening knowledge on the relation between hydration status, water intake and body composition are of interest. Until recently, the assessment of hydration status as the estimation of water intake has involved great difficulty [35,36] as there is no “gold standard” and the techniques and methods available are expensive, invasive and/or complex, making them difficult to apply at the population level [35,36,37,38,39]. Nevertheless, recently, our research group designed a questionnaire that has demonstrated validity in achieving the previous objective and which has been validated in a healthy adult population [40], the hydration status questionnaire (HSQ), and in a healthy adolescent population [41], the hydration status questionnaire for adolescent young (HSQ-AY). In the present study, our aim was to analyze the association of hydration status estimated by means of water intake and balance obtained through the HSQ-AY with body composition and body weight in a population of adolescents from Spain. 

## 2. Materials and Methods

This school-based cross-sectional study was carried out in several periods between the years 2018 and 2019. Volunteers were recruited from a total of seven state and private schools in several cities in Spain—specifically, in Madrid (average temperature of 15.8 °C in October 2018 and an average temperature of 6.8 °C in January 2019), in Victoria (average temperature of 1.3 °C in February 2019), in Murcia (average temperature of 10.8 °C in February 2019) and in Alicante (average temperature of 14.0 °C in March 2019). The inclusion criteria were individuals who were (a) mentally and physically healthy and (b) aged 12–18 years. Exclusion criteria were (a) individuals suffering from diseases related to hydration status, including renal impairment, urinary tract infection, water balance disease and diabetes and/or (b) females who were menstruating during the study. Volunteer recruitment was performed through informative talks in each school. 

Ethical approval was granted by the Clinical Research Ethics Committee of the CEU San Pablo University (Madrid). The corresponding ethical code was 120/16/06, and the study was performed in accordance with the ethical standards laid down in the 1964 Declaration of Helsinki and its later amendments. Participants were informed about the objectives of the study and the procedures involved and signed an informed consent prior to their inclusion in the study. All personal data were confidential and only investigators assigned to the project had access to them, complying with the General Data Protection Regulation (2016/679) and with the Personal Data protection Act of 2018. 2.1 Study Protocol.

### 2.1. Study Protocol

**Figure 1:**
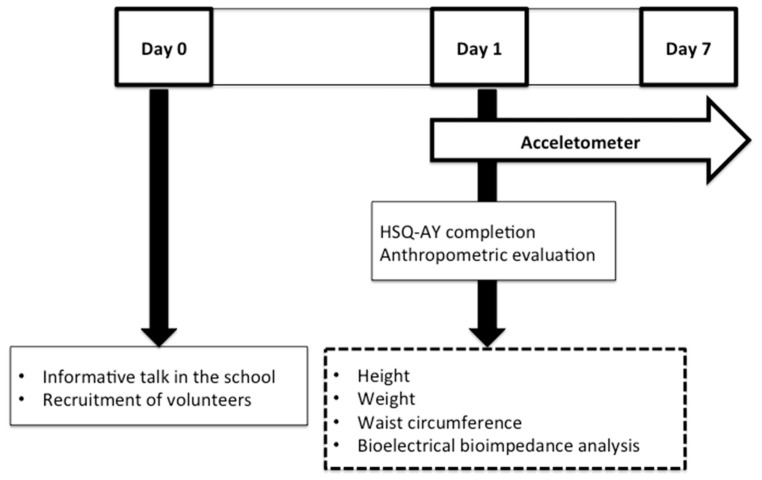
Protocol of the study. HSQ-AY: hydration status questionnaire in a healthy adolescent young Spanish population.

The protocol of the study (Figure 1) was explained to the potential volunteers prior to their inclusion.

The study involved water intake and hydration status assessment, anthropometric evaluation and physical activity assessment of the subjects. Participants completed the HSQ-AY [41], previously validated in a healthy adolescent population, which allowed the estimation of water intake from foods and beverages and water elimination from urine, feces and sweat. In particular, the variables: drinking water (which included tap water and bottle water, still and sparkle), water from beverages (drinking water and water from juices, sodas, milk and dairy products, coffees, tea and infusions, alcoholic beverages and other beverages), water from food (vegetables, fruits and cooked dishes with high water content such as soups), water intake defined as the sum of water from beverages and water from food, and water balance defined as the net difference between water intake and water elimination, were analyzed. Volunteers also wore an accelerometer during seven consecutive days, which estimated not only the quantity of physical activity performed but also its intensity [42]. This information combined with the HSQ-AY was used to estimate water elimination by sweat. Water balance was calculated as the difference between total water intake and total water elimination. 

The anthropometric evaluation comprised the measurement of weight by a digital scale with an accuracy of 200 g (SECA^TM^ 877), height, measured to the nearest 0.1 cm using a wall-mounted stadiometer (SECA^TM^), and waist circumference, which was measured with a flexible tape (Cescorf™). Anthropometric measurements were performed according to the recommendations of the International Standards for Anthropometric Assessment (ISAK) [43] by level I and II accredited anthropometrists. Weight and height data were used to calculate the body mass index (BMI), or Quetelet index, according to the following formula [44]:

BMI = weight (Kg)/height^2^ (m))

Subjects were classified as underweight, normal weight, overweight and obese according to the percentiles established by the Faustino Orbegozo Foundation (Spanish BMI cut-offs) [45] for sex and age. Finally, body composition (total body water, fat body mass, lean body mass, and dry lean body mass) was estimated by bioelectrical impedance (BIA) with a Bioscan Spectrum Multifrequency^TM^. Multi-frequency bioelectrical impedance analysis is described as a tool able to assess total, extracellular and intracellular fluid in humans and seems to be a more accurate method for estimating the TBW compartment for healthy and obese adults [46,47,48,49]. Specifically, Bioscan Spectrum Multifrequency^TM^ works by measuring the impedance at two frequencies of 5 and 50 kHz with an accuracy of impedance of 2–3 Ω, a reactance (50 kHz) of ±1 Ω and a phase angle (50 kHz) of ±0.2.This test was performed under controlled conditions [50]: with participants in fasting conditions (liquid and solid), without practicing intense physical activity in the previous 24 h, with the recommended body posture (lying down) and correct electrode positioning, and in the same time-slot (from 09:00 to 10:00). 

### 2.2. Statistical Analysis

Results are presented as median and interquartile range. Variables were tested for normality using the Shapiro–Wilk test. Differences between variables were assessed with the Mann–Whitney U test and considered significant at *p* ≤ 0.05. The population study was classified by gender and the statistical analysis was performed in each group independently given their physiological differences with respect to their anthropometric characteristics.

The relation between hydration status, water intake (drinking water, water from beverages, water from food, and total water intake) and anthropometric characteristics (weight, BMI, waist circumference, fat body mass, lean body mass, dry lean body mass, and total body water) was evaluated using Spearman (Rho) correlation coefficient. Differences in water consumption and hydration status in relation to BMI were analyzed by the Kruskal–Wallis test followed by the Dunn test to adjust for multiple comparison and adjust *p* values with Bonferroni correction were applied. Finally, differences in anthropometric and body composition variables according to water balance percentiles (< *p*25 = −1545.9 mL, *p*25–*p*50 = −1545.9 to −835.7 mL, *p*50–*p*75 = −835.6 to −145.4 mL, > *p*75 = −145.4 mL) and according to water intake normalized by body weight percentiles (< *p*25 = 40.7 mL/Kg, *p*25–*p*50 = 40.7 – 53.0 mL/Kg, *p*50–*p*75 = 53.1–69.5 mL/Kg, > *p*75 = 69.5 mL/Kg) were also analyzed by the Kruskal–Wallis test followed by the Dunn test to adjust for multiple comparison and adjust *p* values with Bonferroni correction. A linear regression model was constructed to explore the associations between water intake adjusted by body weight as a dependent variable and body composition variables as independent predictors. All statistical analyses were performed using SPSS 24.0 Software (IBM Corp., Armonk, NY, USA). 

## 3. Results

### 3.1. Sample Characteristics 

**Figure 2:**
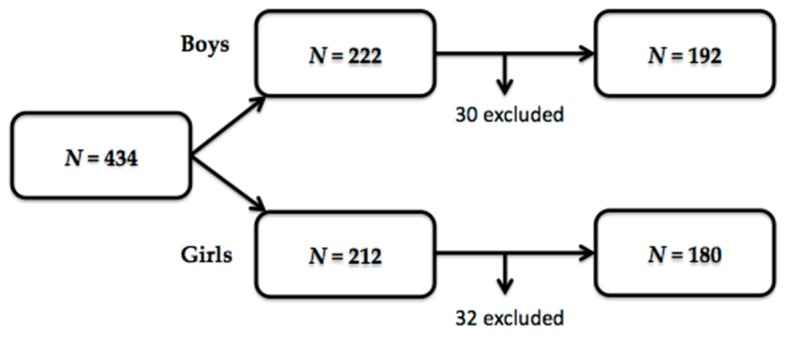
Effective sample size.

A total of 434 volunteers (222 boys and 212 girls) were recruited for the study. In total, 62 of them were excluded after meeting some of the exclusion criteria or not adequately accomplishing a part of the study. Therefore, 372 healthy volunteers—192 boys (51.6%) and 180 girls (48.4%)—were included in the data processing (Figure 2). 

Their anthropometric characteristics are presented in Table 1, where it can be observed that there were significant differences between genders in most of the collected variables although no significant differences were found for weight, height and BMI. 

In the studied population, and according to the BMI percentile, overweight and obesity prevalence was 13.7% and data analyzed by gender showed that prevalence was higher in boys when compared to girls (17.2% and 10.0%, respectively). Nevertheless, the prevalence of underweight for the total sample was 5.6%—higher in girls (6.1%) than in boys (5.2%). Finally, 80.6% of the sample presented normal weight (boys: 77.6%; girls: 83.9%). 

Results of the HSQ-AY sorted by gender are presented in Table 2. We found no differences in water intake variables between genders. Nevertheless, water balance was significantly higher in girls and conversely, total water elimination was significantly higher in boys. In particular, water elimination from feces and sweat was higher in boys (water elimination from feces: Boys (B): 150.0 (131.3–150.0) mL; girls (G): 131.2 (112.5–150.0) mL *p* = 0.002, water elimination from sweat: B: 2268.3 (1790.3–2869.8) mL, G: 1634.9 (1299.3–2049.4); *p* = 0.000). Water elimination from urine was higher in girls than in boys (G: 1222.2 (1187.5–1625.0) mL; B: 1187.5 (1187.5.9–1635.0) mL *p* = 0.000). 

### 3.2. Hydration Status and Body Composition

Associations between water intake from all the analyzed sources and water balance in boys and girls are presented in Table 3; Table 4**,** respectively. 

It can be observed that water balance was inversely correlated with BMI percentile in both boys and girls, confirming the relation between hydration status and body weight. In addition, in boys, it was also inversely correlated with waist circumference, fat body mass and positively with total body water. Although these associations were weak, they confirm the relation between hydration status and body composition. 

Regarding body composition variables, in boys, total body water was positively correlated with water from beverages, total water intake and water balance and in contrast, fat body mass was inversely correlated with the same variables. No correlations were found between water intake and body composition variables in girls. It is important to take into account that the effect size of the mentioned associations was weak. 

Nevertheless, after data normalization by body weight (Table 5 and Table 6), associations between water intake variables from all sources with the majority of anthropometric parameters were found in both genders. In addition, when water intake variables were normalized by body weight, the effect size of the associations found was stronger than when absolutes values of water intake were studied. 

In boys, a stronger inverse association was found between total water intake, weight and BMI. In addition, inverse moderate associations between this variable and waist circumference, fat body mass, dry lean body mass and liters of total body water were determined, whereas positive associations of the same variables with total body water percentage were found. As can be observed, drinking water, water from beverages and water from food normalized by body weight were associated in the same direction with anthropometric variables, but the effect size of the association was less strong. Regarding girls, inverse moderate associations were found between total water intake and weight, BMI, dry lean body mass and fat body mass (Kg) Inverse weak associations were found between this variable and waist circumference, fat body mass (%), lean body mass and total body water (L), and positive weak association with total body water (%). Similar associations were found between drinking water, water from beverages and water from food, with the anthropometric variables although the size of these associations was slighter.

Differences in water intake variables and water balance related to BMI in boys and girls are presented in Table 7 and Table 8**,** respectively. In boys, significant differences were found in water balance of normal weight participants when compared to overweight/obese ones, who presented lower water balance. Significant differences were also found in drinking water, water from beverages, water from food and total water intake normalized by body weight in both boys and girls, showing a decline in water consumption as subjects’ BMI increases. 

Differences in anthropometric and body composition variables related to water balance percentiles were analyzed and significant differences in BMI, fat body mass and waist circumference were found in boys (Table 9). Noteworthy, BMI as well as fat body mass and waist circumference were significantly higher in the lowest percentile of water balance. No differences, however, were found in girls. 

Finally, differences in body composition and anthropometric variables related to water intake normalized by body weight were also analyzed. Results obtained in boys and girls are presented in Table 10 and Table 11. In both genders there were significant differences in body weight, BMI, fat body mass and waist circumference according to water consumption, being higher in the lower percentiles of water intake normalized by body weight. In addition, body water content was significantly higher in the highest percentiles of water intake normalized by body weight. 

To evaluate the independent relation between water intake adjusted by body weight and body composition variables (waist circumference, fat body mass and dry lean body mass), a linear regression model was used (Table 12). The water intake adjusted by body weight was related significantly with fat body mass and dry lean body mass (both, *p* = 0.000).

## 4. Discussion

The significant increase in overweight and obesity prevalence makes research focused on elucidating the causes of these pathologies especially necessary and challenged. In children and adolescent populations, the management of these diseases is especially crucial in order to prevent associated diseases such as type 2 diabetes mellitus, cardiovascular disease, some cancers, kidney disease, obstructive sleep apnea, gout, osteoarthritis, and hepatobiliary disease, among others [51]. It is well known that both overweight and obesity have a multifactorial etiology including genetic, environmental and behavioral factors [51]. In this context, recently, the important role of hydration in health and wellness [12,52,53] has received great attention in research, and several investigations have demonstrated the beneficial effects of water consumption in weight management [17,19]. Nevertheless, little is known about its potential effect on body composition as well as the effect of hydration status in these same parameters [54,55,56]. 

Our present results confirm the relation between water intake from different sources with body weight and with body composition among a sample of healthy Spanish adolescents. From our knowledge, this is the first time that a study confirms the existence of this association in the target population. Interestingly, the fact that higher levels of water balance and water consumption were associated with lower BMI and with a healthier body composition is of great importance, given that it could be useful in the prevention of overweight and obesity and confirm the existence of an important relation between hydration status, body weight and body composition. When specific water sources and water balance were studied against anthropometric and body composition variables, we found that water balance was not only inversely correlated with BMI percentiles in both boys and girls, but also, in boys, it was inversely correlated with relevant variables such as waist circumference and fat body mass, and positively associated with total body water. Furthermore, a positive correlation between water intake and total body water and an inverse association with fat body mass was observed mainly in boys. Regarding girls, we found that water from beverages as well as total water intake were inversely correlated with BMI percentile, but no correlations were obtained for the rest of the parameters. It is important to mention that the effect size of the associations found was weak and future investigations which confirm these results are of interest. These differences between genders could be explained by the anthropometric changes associated with pubertal development, with an earlier start in girls (8–13 years of age) than in boys (9–14 years of age) [57], and which implies great differences in anthropometric characteristics between individuals of the same age, giving rise to a very heterogeneous girls group.

Nowadays, water is considered an essential nutrient [33] and as consequence, it should be regarded as such. Because of that, given the important role of body size in nutrients requirements [58], in the current study, water consumption was normalized by body weight, as an approach to body size. Its relationship with body composition and weight status was also investigated, observing differences relating to the absolute values of water intake. The main differences affect, first of all, the number of associations found. In boys, absolute values of water consumption were positively associated with total body water, and inversely with fat body mass while in girls, an inverse association with BMI percentiles was found. Nevertheless, when body weight was considered, all the anthropometric variables were associated with water consumption in boys (drinking water, water from beverages, water from food and water intake were positively correlated with total body water percentage and inversely with the rest of the parameters: weight, BMI, waist circumference, fat body mass, lean body mass, dry lean body mass, and liters of total body water). In girls, correlations were found with the majority of them (except for drinking water, which was not correlated with total body water percentage and fat body mass and water from food, which was not associated with liters of total body water, lean body mass and dry lean body mass). Surprisingly, the direction of certain associations also changed. In particular, those variables that depend on body size (lean body mass, dry lean body mass and liters of total body water) were associated inversely with water intake normalized by body weight. These changes in the direction of the associations are due to a higher body size, which necessarily involves a higher amount of these components, but not a higher proportion or relative amount of them. Lastly, when water intake variables were normalized by body weight, the associations found with anthropometric variables were stronger than when absolute values of water intake were studied. These findings are in accordance with the results recently published by our group in healthy adults [21]. Likewise, linear regression analysis showed that BIA measures such as fat body mass and dry lean body mass were independent predictors of water intake normalized by body weight in the study population. These findings suggest that a suitable water intake could improve body composition. Nevertheless, waist circumference was not found to be a significant predictor of water intake normalized by body weight in the linear regression analysis. Thus, in addition, body composition parameters should be taken into account in hydration status monitoring. Therefore, a “tailored water prescription” that considers gender, age and body composition differences is required.

To analyze the obtained results further, the study population was categorized by BMI. Differences found in water balance and water consumption are of interest, because they confirm and strengthen the previous results mentioned: water intake normalized by body weight was higher in normal-weight and underweight individuals when compared to overweight/obese individuals. Furthermore, water balance was higher in normal-weight boys than in those with excess weight. Additionally, individuals were also categorized by percentiles of water intake normalized by body weight, and it has been observed that those individuals who were within the higher percentiles had a better weight status and body composition (lower fat body mass, weigh and waist circumference, and higher total body water) than those who were in the lowest water intake percentiles. Again, we presented similar findings among healthy adult subjects [21].

These facts bring to light the important role of body size in water requirements. Additionally, other intrinsic (gender) and extrinsic (physical activity, drugs consumption, energy intake, environmental temperature and humidity) factors may affect water needs [59,60,61,62] and consequently, water intake recommendations should be as personalized as much as possible. In spite of this, to date, there are no water intake recommendations per body weight in children and adolescents, although it has already been established for free-living adults [63,64]. The EFSA [32] recommends a daily water intake for children from 9 to 13 years of 2100 mL in boys and 1900 mL in girls. Adolescents of 14 years and more are considered adults with respect to water intake recommendations (females: 2000 mL and males: 2500 mL). In the current study, both girls (water intake: 2731.9 (2575.5–2888.2) mL/day) and boys (water intake: 2869.1 (2721.7–3016.4) mL/day) fulfilled the EFSA water intake recommendations when all the sources of water intake (drinking water, water from beverages and water from food) were considered. Mean water intake values were similar between genders, which was in agreement with available literature [65]. However, water balance was significantly higher among girls, a fact that can be explained by the higher water elimination values (via feces and sweat) obtained in boys. The higher water losses by sweating were mainly due to the intensity and duration of the physical activity practiced, which was estimated through accelerometers, considered as the most refined method to quantify physical activity [42]. On the other hand, water consumption obtained in the current study was slightly higher when compared to other investigations [65,66]. These differences could be mainly due to the methodology used for data collection since most of these studies used unspecific questionnaires for water intake estimation, which could lead to underestimation, as they solely record water consumption in the main meals, dismissing the fluid intake that may occur throughout the day [67,68]. In the present study, the recently validated questionnaire “HSQ-AY” [41], which was specifically designed to recall water consumption, elimination and water balance was used. On the other hand, the fact that participation in the study was entirely voluntary and that the anthropometric measurement collection may have constituted a barrier in certain population groups could be influencing the overweight and obesity prevalence of the population analyzed, which was lower than the available data for the general population [3]. 

The strengths of the present study include the novelty, the important associations found between the hydration status (HS) and body composition, the use of a specific validated hydration questionnaire, the quality of the anthropometric data collected and the sample size. However, the current study is not exempt of limitations. The most important weaknesses refer to the impossibility of directly assessing water consumption as well as body composition, which have been estimated through a validated questionnaire and BIA respectively. Nevertheless, given the ethical and economic implications of direct measurement, it is impossible to apply them to the general population. Another limitation is the impossibility of the designed study to differentiate the effect of each type of beverage on body weight and body composition and the lack of sex hormone level information, which could explain the differences found between genders. In addition, underreporting and misreporting of water and other beverages and food intakes by subjects could have an influence in overall water intake and balance and, according to literature, women are more likely to underreport than men [69]. Nevertheless, in the present study, underreporting and misreporting was not assessed. Lastly, although it is known that most of the participants belong to a private or state school, the socioeconomic status of their families was not assessed. 

## 5. Conclusions

Our results demonstrate that there is a clear association between hydration status and fluid intake with body composition and body weight. Therefore, further research into individualized water intake and water balance strategies could be useful in weight management and overweight and obesity prevention amongst healthy young adolescents. 

## Figures and Tables

**Table 1:** Anthropometric characteristics of participants from the study.

Anthropometric Variables	Boys (*n* = 192)	Girls (*n* = 180)	*p* Values
Age	13.0 (12.0−14.0)	13.0 (12.0–14.0)	0.427
Weight (Kg)	49.0 (43.4−59.0)	51.0 (45.4−54.8)	0.507
Height (cm)	160.2 (154.2−166.3)	160.1 (155.1−164.5)	0.536
BMI (Kg/m^2^)	18.8 (17.5−21.3)	19.4 (17.8−21.2)	0.201
WC (cm)	66.0 (63.0− 70.9)	63.5 (60.1−66.6)	0.000
TBW (%)	56.2 (52.8−60.0)	53.2 (50.2−56.2)	0.000
TBW (L)	28.1 (24.5−32.3)	27.1 (24.7−29.2)	0.005
FBM (%)	24.6 (20.0−29.4)	29.6 (26.2−33.9)	0.000
FBM (Kg)	12.0 (9.3−16.1)	15.3 (12.0−18.6)	0.000
LBM (Kg)	37.5 (32.5−42.9.)	35.5 (32.7−38.6)	0.001
DLBM (Kg)	9.5 (8.1−10.9)	8.6 (7.8−9.4)	0.000

Results are presented as the median and interquartile range; *p* values derived through the Mann–Whitney U test. (BMI: Body Mass Index, WC: waist circumference, TBW: total body water, FBM: fat body mass, LBM: lean body mass, and DLBM: dry lean body mass).

**Table 2:** Water intake from all sources, water elimination and water balance of participants from the study, sorted by gender.

	Boys (*n* = 192)	Girls (*n* = 180)	*p* Values
Drinking water (mL/day)	1650.0	1400.0	
	(1000.0–2000.0)	(1000.0–2000.0)	0.089
Water from beverages (mL/day)	2339.1	2086.6	
	(1779.0–2890.2)	(1575.7–2769.7)	0.067
Water from food (mL/day)	383.5	413.5	
	(257.8–544.4)	(265.7–596.4)	0.227
Water intake (mL/day)	2648.8	2509.5	
	(2217.4–3352.7)	(1923.9–3315.9)	0.153
Total water loss (mL/day)	3734.7	3172.7	
	(3183.6–4327.4)	(2746.1–3785.3)	0.000
Water balance (mL/day)	−979.3	−661.0	
	(−1748.8 to −308.1)	(−1329.2 to 160.1)	0.002
Drinking water /weight (mL/Kg)	32.2	28.4	
	(21.7–42.6)	(19.1–41.2)	0.122
Water from beverages/weight (mL/ Kg)	46.3	41.8	
	(35.1–61.1)	(30.9–56.0)	0.051
Water from food/weigh (mL/Kg)	7.6	8.1	
	(4.7–11.4)	(5.4–12.6)	0.369
Water intake/weight (mL/Kg)	53.9	50.7	
	(42.6–70.4)	(38.6–66.1)	0.135

Results are presented as median and interquartile range; *p* values derived through Mann–Whitney U test.

**Table 3:** Correlation between water intake and balance with anthropometric and body composition variables in boys.

	Drinking Water (mL/day)		Water from Beverages (mL/day)		Water from Food (mL/day)		Water Intake (mL/day)		Water Balance (mL/day)	
	*r*	*p* Values	*r*	*p* Values	*r*	*p* Values	*r*	*p* Values	*r*	*p* Values
Weight (Kg)	0.051	0.485	0.026	0.718	−0.141	0.050	0.021	0.777	−0.060	0.406
BMI (Kg/m^2^)	−0.048	0.512	−0.093	0.197	−0.187	0.009	−0.099	0.171	−0.206	0.004
BMI p (Kg/m^2^)	−0.085	0.242	−0.091	0.209	−0.120	0.098	−0.076	0.294	−0.225	0.002
WC (cm)	−0.088	0.225	−0.085	0.241	−0.091	0.211	−0.073	0.317	−0.183	0.011
TBW (%)	0.115	0.113	0.187	0.009	0.118	0.104	0.174	0.016	0.158	0.029
TBW (L)	0.131	0.070	0.139	0.055	−0.117	0.107	0.119	0.099	0.035	0.632
FBM (%)	−0.124	0.088	−0.195	0.007	−0.116	0.109	−0.182	0.011	−0.167	0.021
FBM (Kg)	−0.043	0.555	−0.100	0.167	−0.147	0.042	−0.094	0.195	−0.104	0.152
LBM (Kg)	0.111	0.125	0.120	0.098	−0.122	0.092	0.101	0.162	0.038	0.601
DLBM (Kg)	0.129	0.075	0.136	0.060	−0.110	0.127	0.118	0.104	0.037	0.607

Results are presented as Spearman’s (Rho) correlation coefficient. (BMI: body mass index, BMI p: body mass index percentiles, WC: waist circumference, TBW: total body water, FBM: fat body mass, LBM: lean body mass, and DLBM: dry lean body mass).

**Table 4:** Correlation between water intake and balance with anthropometric and body composition variables in girls.

	Drinking Water (mL/day)		Water from Beverages (mL/day)		Water from Food (mL/day)		Water Intake (mL/day)		Water Balance (mL/day)	
	r	*p* Values	r	*p* Values	r	*p* Values	r	*p* Values	r	*p* Values
Weight (Kg)	0.039	0.606	0.013	0.868	0.094	0.212	0.025	0741	0.023	0.762
BMI (Kg/m^2^)	0.020	0792	−0.045	0.553	−0.041	0.584	−0.059	0.431	−0.025	0.737
BMI p (Kg/m^2^)	−0.081	0282	−0.173	0.020	−0.094	0.211	−0.180	0.015	−0.180	0.016
WC (cm)	0.034	0.653	0.006	0.934	0.009	0.904	−0.003	0.968	−0.119	0.112
TBW (%)	−0.061	0.413	−0.023	0.762	0.051	0.498	0.013	0.860	−0.049	0.517
TBW (L)	0.007	0.927	−0.003	0.973	0.139	0.062	0.029	0.702	−0.016	0.836
FBM (%)	0.077	0.304	0.020	0.786	−0.032	0.672	−0.007	0.925	0.045	0.546
FBM (Kg)	0.079	0.292	0.028	0.707	0.012	0.877	0.010	0.890	0.047	0.533
LBM (Kg)	−0.013	0.862	−0.020	0.792	0.127	0.088	0.010	0.898	−0.018	0.809
DLBM (Kg)	−0.034	0.648	−0.038	0.611	0.110	0.140	−0.012	0.875	0.010	0.893

Results are presented as Spearman’s (Rho) correlation coefficient. (BMI: body mass index, BMI p: body mass index percentiles, WC: waist circumference, TBW: total body water, FBM: fat body mass, LBM: lean body mass, and DLBM: dry lean body mass).

**Table 5:** Correlation between water intakes normalized by body weight with anthropometric and body composition variables in boys.

	Drinking Water/Weight (mL/day/Kg)		Water from Beverages/Weight (mL/day/Kg)		Water from Food/Weight (mL/day/Kg)		Water Intake/Weight (mL/day/Kg)	
	*r*	*p* Values	*r*	*p* Values	*r*	*p* Values	*r*	*p* Values
BMI (Kg/m^2^)	−0.388	0.000	−0.497	0.000	−0.440	0.000	−0.548	0.000
BMI p (Kg/m^2^)	−0.320	0.000	−0.368	0.000	−0.299	0.000	−0.395	0.000
WC (cm)	−0.390	0.000	−0.447	0.000	−0.328	0.000	−0.476	0.000
TBW (%)	0.250	0.000	0.332	0.000	0.213	0.003	0.342	0.000
TBW (L)	−0.239	0.001	−0.319	0.000	−0.395	0.000	−0.379	0.000
FBM (%)	−0.243	0.001	−0.321	0.000	−0.200	0.006	−0.329	0.000
FBM (Kg)	−0.340	0.000	−0.454	0.000	−0.367	0.000	−0.488	0.000
LBM (Kg)	−0.255	0.000	−0.332	0.000	−0.394	0.000	−0.389	0.000
DLBM (Kg)	−0.235	0.001	−0.314	0.000	−0.386	0.000	−0.372	0.000

Results are presented as Spearman’s (Rho) correlation coefficient. (BMI: body mass index, BMI p: body mass index percentiles, WC: waist circumference, TBW: total body water, FBM: fat body mass, LBM: lean body mass, and DLBM: dry lean body mass).

**Table 6:** Correlation between water intakes normalized by body weight with anthropometric and body composition variables in girls.

	Drinking Water/Weight (mL/day/Kg)		Water from Beverages/Weight (mL/day/Kg)		Water from Food/Weight (mL/day/Kg)		Water Intake/Weight (mL/day/Kg)	
	*r*	*p* Values	*r*	*p* Values	*r*	*p* Values	*r*	*p* Values
BMI (Kg/m^2^)	−0.254	0.000	−0.348	0.000	−0.273	0.000	−0.376	0.000
BMI p (Kg/m^2^)	−0.281	0.000	−0.386	0.000	−0.271	0.000	−0.400	0.000
WC (cm)	−0.178	0.017	−0.233	0.002	−0.181	0.015	−0.253	0.001
TBW (%)	0.125	0.096	0.197	0.008	0.214	0.004	0.237	0.001
TBW (L)	−0.247	0.001	−0.294	0.000	−0.099	0.184	−0.274	0.000
FBM (%)	−0.117	0.116	−0.207	0.005	−0.198	0.008	−0.242	0.001
FBM (Kg)	−0.181	0.015	−0.270	0.000	−0.216	0.004	−0.300	0.000
LBM (Kg)	−0.275	0.000	−0.318	0.000	−0.118	0.116	−0.299	0.000
DLBM (Kg)	−0.296	0.000	−0.336	0.000	−0.135	0.071	−0.319	0.000

Results are presented as Spearman’s (Rho) correlation coefficient. (BMI: body mass index, BMI p: body mass index percentiles, WC: waist circumference, TBW: total body water, FBM: fat body mass, LBM: lean body mass, and DLBM: dry lean body mass).

**Table 7:** Differences in water intake variables and water balance according to body mass index among boys.

	Underweight (*N* = 10)	Normal Weight (*N* = 149)	Overweight/Obesity (*N* = 33)
Drinking water (mL/day)	1575.0	1650.0	1600.0
	(1187.5–2392.5)	(1000.0–2050.0)	(1000.0–1890.0)
Water from beverages (mL/day)	2390.7	2063.1
	(1717.3–2892.8)	(1869.8–2924.7)	(1455.7–2566.5)
Water from food (mL/day)	531.0	382.7	321.0
	(399.2–707.2)	(253.2–525.2)	(230.6–518.6)
Water intake (mL/day)	2315.5	2690.6	2584.8
	(2103.6–3473.9)	(2273.8–3455.9)	(2040.0–3112.5)
Water balance (mL/day)	−931.1^ab^	−946.5^a^	−1522.1^b^
	(−1219.7 to −308.1)	(−1584.9 to −274.4)	(−2085.1 to −734.2)
Drinking water/ body weight (mL/Kg)	39.3^c^	35.4^c^	23.2^d^
	(30.5–70.1)	(22.8–44.2)	(15.8–28.4)
Water from beverages/body weight (mL/Kg)	50.6^e^	48.6^e^	31.1^f^
	(39.4–84.4)	(38.2–62.2)	(23.1–40.8)
Water from food/body weight (mL/Kg)	14.1^g^	8.1^h^	5.0^i^
	(9.8–19.7)	(5.1–11.4)	(3.5–9.2)
Water intake/body weight (mL/Kg)	64.7^j^	55.7^j^	40.2^k^
	(52.1–101.3)	(45.9–75.5)	(30.0–50.7)

Data reported as median and interquartile range per group. Different superscript lowercase letters indicate statistical significance in each row (*p* ≤ 0.05) assessed using the Kruskal–Wallis test followed by the Dunn test to adjust for multiple comparisons and adjust the *p* value with Bonferroni correction. Body mass index percentile according to Orbegozo criteria.

**Table 8:** Differences in water intake variables and water balance according to body mass index among girls.

	Underweight (*N* = 11)	Normal Weight (*N* = 151)	Overweight/Obesity (*N* = 18)
Drinking water (mL/day)	1800.0	1400.0	1550.0
	(1320.0–2000.0)	(1000.0–2000.0)	(950.0–1985.0)
Water from beverages (mL/day)	2651.0	2065.6	1898.6
	(1979.5–3373.0)	(1571.0–2771.3)	(1265.9–2531.8)
Water from food (mL/day)	488.5	400.8	400.4
	(387.1–729.1)	(262.4–601.0)	(203.5–494.1)
Water intake (mL/day)	3219.7	2490.8	2551.4
	(2498.7–3913.1)	(1912.2–3393.4)	(1807.0–2921.8)
Water balance (mL/day)	−540.3	−664.1	−933.3
	(−578.8 to −984.5)	(−1350.6–196.7)	(−1583.8 to −457.8)
Drinking water/ body weight (mL/Kg)	46.5^a^	28.1^b^	23.1^b^
	(37.9–58.6)	(19.0–41.1)	(16.6–29.9)
Water from beverages/body weight (mL/Kg)	67.4^c^	42.1^d^	29.7^e^
	(53.9–86.5)	(31.8–55.4)	(19.5–39.2)
Water from food/body weight (mL/Kg)	14.8^f^	8.1f^g^	5.8^g^
	(10.6–15.9)	(5.4–11.7)	(3.1–8.1)
Water intake/body weight (mL/Kg)	82.4^h^	52.1^i^	38.6^j^
	(67.5–104.4)	(39.4–65.7)	(26.5–46.5)

Data reported as median and interquartile range per group. Different superscript lowercase letters indicate statistical significance in each row (*p* ≤ 0.05) assessed using the Kruskal–Wallis test followed by the Dunn test to adjust for multiple comparisons and adjust the *p* value with Bonferroni correction. Body mass index percentile according to Orbegozo criteria.

**Table 9:** Differences in anthropometric and body composition variables according to water balance percentiles among boys.

Percentiles	Distribution of Water Balance (mL)			
	< *p* 25 (*N* = 58)	*p* 25–p50 (*N* = 50)	*p* 50–*p*75 (*N* = 47)	> *p* 75 (*N* = 37)
Weight (Kg)	50.1	47.3	51.0	46.6
	(44.4–61.8)	(42.4–56.7)	(45.6–60.1)	(42.0–55.8)
BMI (Kg/m^2^)	19.9^a^	18.6^ab^	19.2^a^	18.0^b^
	(17.9–22.6)	(17.6–21.0)	(18.0–21.4)	(16.6–19.6)
FBM (%)	27.5^c^	22.9^cd^	25.0^cd^	21.5^d^
	(21.9–32.5)	(19.3–28.6)	(21.5–29.4)	(18.8–26.8)
FBM (Kg)	13.2	10.6	12.9	10.4
	(10.0–18.7)	(8.1–14.5)	(10.5–16.1)	(8.7–15.5)
WC (Cm)	68.0^e^	64.8^ef^	67.4^e^	63.8^f^
	(63.9–74.0)	(62.4–69.0)	(63.9–72.2)	(61.0–67.0)
LBM (Kg)	37.3	36.1	39.7	36.7
	(32.3–44.4)	(32.4–40.9)	(33.2–42.8)	(30.8–45.4)
DLBM (kg)	9.3	9.3	9.9	9.3
	(7.9–11.2)	(8.0–10.4)	(8.4–10.8)	(7.8–11.6)
TBW (%)	54.2	58.1	55.9	58.2
	(50.6–58.7)	(53.6–60.7)	(53.1–58.7)	(54.8–60.9)

Data reported as median and interquartile range per group. Different superscript lowercase letters indicate statistical significance in each row (*p* ≤ 0.05) according to the Kruskal–Wallis test followed by the Dunn test to adjust for multiple comparisons and adjust the *p* value with Bonferroni correction. Percentiles of water balance: < *p*25 = −1545.9 mL; *p*25–*p*50 = −1545.9 to −835.7 mL; *p*50–*p*75 = −835.7 to −145.4 mL; >*p*75 = −145.3 mL (*p*: percentiles, BMI: body mass index, WC: waist circumference, TBW: total body water, FBM: fat body mass, LBM: lean body mass, and DLBM: dry lean body mass).

**Table 10:** Differences in anthropometric and body composition variables according to water intake normalized per body weight percentiles in boys.

Percentiles	Distribution of Water Intake/Body Weight (mL/Kg)			
	< *p* 25	*p* 25–*p*50	*p* 50–*p*75	> *p* 75
	(*N* = 42)	(*N* = 51)	(*N* = 46)	(*N* = 53)
Weight (Kg)	59.8^a^	52.3^a^	46.8^b^	43.8^b^
	(49.0–66.8)	(46.6–60.1)	(43.0–51.5)	(37.5–48.4)
BMI (Kg/m^2^)	21.4^c^	20.1^cd^	18.5^de^	17.4^e^
	(19.5–24.3)	(18.3–21.6)	(17.7–19.9)	(16.6–18.5)
FBM (%)	28.6^f^	24.3^fg^	24.4^g^	21.9^g^
	(24.7–32.9)	(21.4–29.4)	(19.6–29.0)	(19.6–27.7)
FBM (Kg)	16.9^h^	13.3^hi^	11.8^ij^	9.4^j^
	(11.5–21.6)	(10.0–15.8)	(8.8–14.7)	(8.1–11.1)
WC (Cm)	72.5^k^	68.0^l^	64.6^lm^	63.8^m^
	(65.0–80.1)	(64.0–71.8)	(61.5–68.3)	(60.8–66.0)
LBM (Kg)	40.9^n^	41.3^n^	35.7^o^	33.5^o^
	(36.9–46.6)	(34.6–45.1)	(31.5–39.4)	(28.1–38.6)
DLBM (kg)	10.3^p^	10.4^p^	8.9^q^	8.4^q^
	(9.3–11.8)	(8.6–11.5)	(7.9–10.0)	(6.9–9.8)
TBW (%)	53.2^r^	56.3^rs^	56.4^s^	58.9^s^
	(50.1–56.4)	(53.3–58.7)	(53.6–60.3)	(54.5–60.6)

Data reported as median and interquartile range per group. Different superscript lowercase letters indicate statistical significance in each row (*p* ≤ 0.05) using the Kruskal–Wallis test followed by the Dunn test to adjust for multiple comparisons and adjust the *p* value with Bonferroni correction. Percentiles of water intake/body weight: < *p*25 = 40.7 mL/Kg; *p*25–*p*50 = 40.8–53.0 mL/Kg; *p*50–*p*75 = 53.1–69.5 mL/Kg; >*p*75 = 69.6 mL/Kg. (*p*: percentiles, BMI: body mass index, WC: waist circumference, TBW: total body water, FBM: fat body mass, LBM: lean body mass, and DLBM: dry lean body mass).

**Table 11:** Differences in anthropometric and body composition variables according to water intake normalized per body weight percentiles in girls.

Percentiles	Distribution of Water Intake/Body Weight (mL/Kg)			
	< *p* 25	*p* 25–*p*50	*p* 50–*p*75	> *p* 75
	(*N* = 51)	(*N* = 43)	(*N* = 47)	(*N* = 39)
Weight (Kg)	53.2^a^	50.9^a^	52.0^a^	45.4^b^
	(48.1–58.0)	(45.5–54.1)	(47.4–54.8)	(40.2–49.7)
BMI (Kg/m^2^)	20.9^c^	19.6^cd^	19.2^de^	17.7^e^
	(19.2–23.1)	(17.8–21.4)	(18.0–21.0)	(16.9–19.9)
FBM (%)	31.4^f^	31.3^f^	29.6^fg^	27.5^g^
	(28.0–35.5)	(27.2–35.6)	(25.0–33.9)	(24.9–29.6)
FBM (Kg)	16.5^h^	15.8^h^	15.1^h^	12.0^i^
	(13.3–21.0)	(13.6–20.5)	(12.0–18.6)	(9.9–16.0)
WC (Cm)	65.9^j^	63.5^jk^	63.2^jk^	60.5^k^
	(61.5–68.0)	(60.8–66.5)	(62.0–66.6)	(57.8–64.0)
LBM (Kg)	37.1^l^	35.1^lm^	36.7^l^	32.9^m^
	(35.1–39.7)	(32.7–38.4)	(33.2–38.9)	(30.0–36.3)
DLBM (kg)	9.0^n^	8.6^no^	8.7^n^	8.0^o^
	(8.3–9.7)	(7.8–9.4)	(7.9–9.3)	(7.2–8.5)
TBW (%)	52.4^p^	52.5^p^	53.4^pq^	55.5^q^
	(49.3–54.5)	(49.0–55.1)	(50.2–56.6)	(53.1–57.5)

Data reported as median and interquartile range per group. Different superscript lowercase letters indicate statistical significance in each row (*p* ≤ 0.05) using the Kruskal–Wallis test followed by the Dunn test to adjust for multiple comparisons and adjust the *p* value with Bonferroni correction. Percentiles of water intake/ body weight: < *p*25 = 40.7 mL/Kg; *p*25–*p*50 = 40.8–53.0 mL/Kg; *p*50–*p*75 = 53.1–69.5 mL/Kg; >*p*75 = 69.6 mL/Kg. (*p*: percentiles, BMI: body mass index, WC: waist circumference, TBW: total body water, FBM: fat body mass, LBM: lean body mass, and DLBM: dry lean body mass).

**Table 12:** Water intake adjusted by body weight in a linear regression analysis.

Variable	B	SEM	β	95%CI	*p* Value
Waist circumference (cm)	−0.244	0.225	−0.070	−0.688 to 0.199	0.279
Fat body mass (%)	−1.042	0.195	−0.284	−1.426 to −0.658	0.000
Dry lean body mass (Kg)	−3.841	0.850	0.274	−5.513 to −2.170	0.000
Constant	136.735	10.946		115.209 to 158.260	

SEM: standard error of the mean, 95%CI: confidence interval, R = 0.429, R2 = 0.184 and R adjusted = 0.177.
